# Dynamin-2 Stabilizes the HIV-1 Fusion Pore with a Low Oligomeric State

**DOI:** 10.1016/j.celrep.2016.12.032

**Published:** 2017-01-10

**Authors:** Daniel M. Jones, Luis A. Alvarez, Rory Nolan, Margarita Ferriz, Raquel Sainz Urruela, Xènia Massana-Muñoz, Hila Novak-Kotzer, Michael L. Dustin, Sergi Padilla-Parra

**Affiliations:** 1 Division of Structural Biology, University of Oxford, The Henry Wellcome Building for Genomic Medicine, Headington, Oxford OX3 7BN, UK; 2 Wellcome Trust Human Genetics, Cellular Imaging Core, University of Oxford, Oxford OX3 7BN, UK; 3 The Kennedy Institute of Rheumatology, University of Oxford, Headington, Oxford OX3 7BN, UK

**Keywords:** HIV-1 fusion, Dynamin-2, advanced light imaging, number and brightness, cell-cell fusion

## Abstract

One of the key research areas surrounding HIV-1 concerns the regulation of the fusion event that occurs between the virus particle and the host cell during entry. Even if it is universally accepted that the large GTPase dynamin-2 is important during HIV-1 entry, its exact role during the first steps of HIV-1 infection is not well characterized. Here, we have utilized a multidisciplinary approach to study the DNM2 role during fusion of HIV-1 in primary resting CD4 T and TZM-bl cells. We have combined advanced light microscopy and functional cell-based assays to experimentally assess the role of dynamin-2 during these processes. Overall, our data suggest that dynamin-2, as a tetramer, might help to establish hemi-fusion and stabilizes the pore during HIV-1 fusion.

## Highlights

• DNM2 is crucial for HIV-1 fusion in T Cells and reporter cells
• DNM2 is not necessarily linked with endocytosis
• DNM2 tetramer stabilizes the HIV-1 fusion pore

## Graphical Abstract

**Figure:**
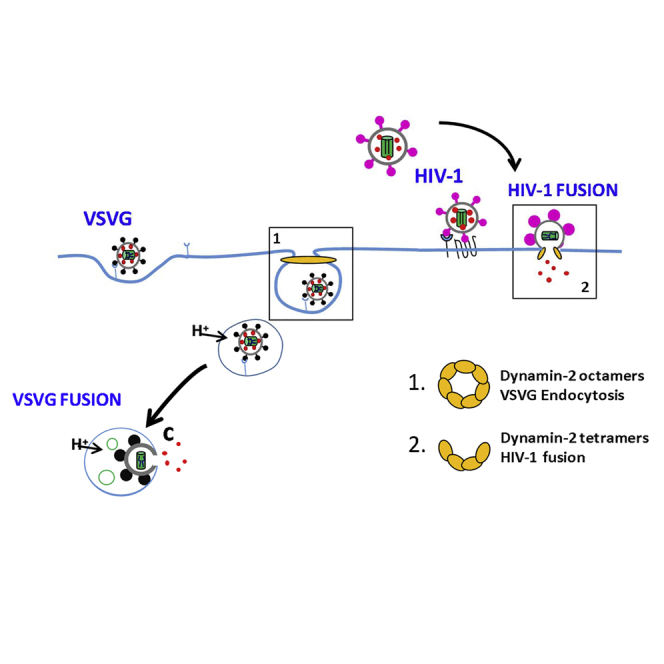

## Teaser

Regulation of HIV-1 fusion is one of the research areas of intense interest. Here, Jones et al. show that the large GTPase dynamin-2 (DNM2) forms a tetramer that acts to stabilize the HIV fusion pore.

## Introduction

One of the key research areas surrounding HIV-1 concerns the regulation of the fusion event that occurs between the virus particle and the host cell during entry. HIV-1 fusion is initiated when conformational alterations to the viral gp120-gp41 envelope proteins occur following binding of the virus to its receptor (CD4) and co-receptor (either CCR5 or CXCR4) (Doms and Trono, 2000), resulting in the release of the viral core into the cytoplasm. Several reports have presented evidence to indicate that HIV-1 fuses directly at the cell membrane in SupT1-R5, CEM-ss and primary CD4 T Cells (Herold et al., 2014). Plasma membrane fusion (Wu and Yoder, 2009) presents a completely different set of challenges for incoming virus particles compared to those entering by post-endocytic fusion (de la Vega et al., 2011, Miyauchi et al., 2009a). For example, fusion events occurring at the plasma membrane mean that incoming particles inevitably encounter an intact cortical actin cytoskeleton, which constitutes a physical barrier that must be overcome for successful infection to occur. As an alternative to plasma membrane fusion, clathrin-mediated endocytosis (CME) allows viruses to cross the cell plasma membrane harbored within endocytic vesicles, followed by a fusion event between the membranes of the virus and the endosome. This process requires precise signaling events to not only initiate the process, but to ensure that fusion occurs prior to degradation of the virus particle within the increasingly toxic environment of the endolysosomal machinery (Stein et al., 1987).

Irrespective of the entry method utilized, it is clear that both the actin rearrangement and dynamin-2 (DNM2) activity are required for successful viral infection to occur (Barrero-Villar et al., 2009, Gordón-Alonso et al., 2013). Interestingly, while several reports clearly show the relevance of DNM2 in HIV-1 fusion (Miyauchi et al., 2009a, Pritschet et al., 2012, Sloan et al., 2013), its exact role during virus entry is yet to be clarified. One of the primary roles of DNM2 is to pinch forming endocytic vesicles from the plasma membrane to yield an endosome during CME (Ferguson and De Camilli, 2012). Thus, the involvement of DNM2 in HIV-1 fusion is incompletely understood since recent evidence indicates that in primary CD4 T cells the virus fuses directly at the plasma membrane and not from within endosomes (Herold et al., 2014), meaning the importance of DNM2 in HIV-1 fusion may be distinct from its role in CME. Here, we have combined advanced light microscopy with cell-based functional assays to recover HIV-1 fusion kinetics for reporter cell lines (TZM-bl) and primary resting CD4 T cells (CXCR4-tropic HXB2) isolated from healthy individuals. Interestingly, the addition of dynasore (a DNM2 inhibitor) at partially inhibitory concentrations (Chou et al., 2014) delayed HIV-1 fusion kinetics in primary CD4 T cells. In addition, we performed fluorescence lifetime imaging microscopy (FLIM) and number and brightness combined with total internal reflection fluorescence microscopy (TIRFM) experiments to ascertain the oligomeric state of DNM2 during HIV-1 fusion. We found that DNM2 adopted a low oligomeric state (a tetramer) when reporter cells (TZM-bl) were exposed to virions with HIV-1 JR-FL envelope proteins. By contrast, cells exposed to HIV-1 virions displaying VSV-G envelope proteins (Env) exhibited higher oligomeric DNM2 states (hexamers and octamers). These data supported insights gained from cell-cell fusion experiments where fusion was delayed by 3–4 min between target cells expressing CD4 and co-receptor (CCR5), and effector cells expressing the HIV-1 envelope were exposed to high concentrations of dynasore. Moreover, we observed flickering of the fusion pore in HIV-1-driven cell-cell fusion experiments when non-inhibitory concentrations of dynasore were used. Collectively, our results suggest that DNM2 might play a critical role inducing hemi-fusion and HIV pore stabilization; probably with a low oligomeric state during fusion pore expansion and dilation within the plasma membrane.

## Results

### Dynasore Inhibits HIV-1 Fusion in Both Reporter TZM-bl Cells and CD4 T Cells

**Figure 1:**
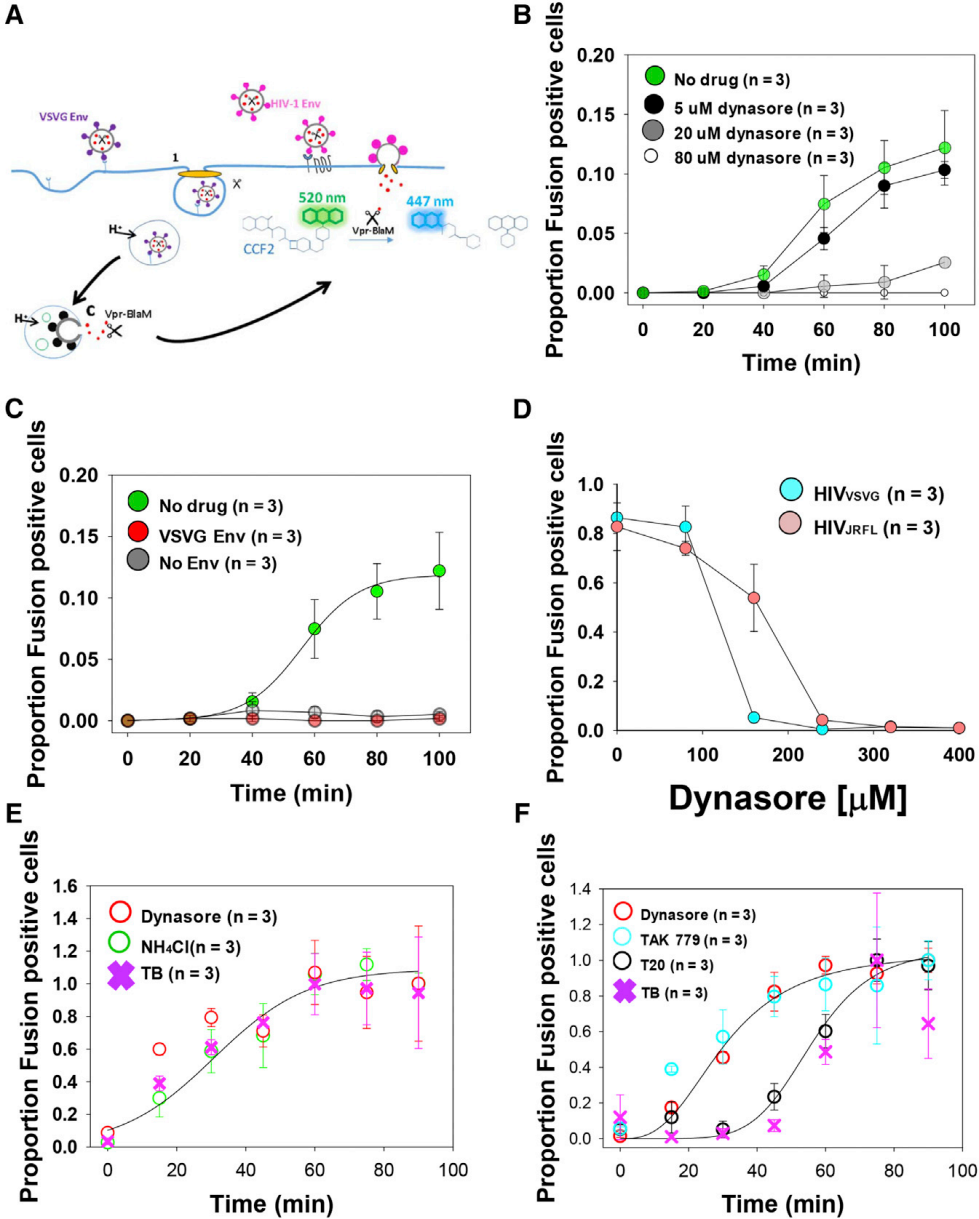
HIV-1 Fusion Kinetics Is Dynamin-2 Dependent in Both CD4 T Cells and TZM-bl Cells (A) Cartoon depicting the BlaM assay. Upon virion fusion and capsid release, the Vpr-BlaM chimera recognizes a FRET reporter (CCF2) that changes color (green to blue) upon cleavage. (B) Real-time BlaM was applied using HIV-1 virions packaging the Vpr-β-Lactamase chimera and pseudotyped with HXB2 Env on primary CD4 T cells at different concentrations of dynasore: 0 μM (green dots), 5 μM (black dots), 20 μM (gray dots), and 80 μM (white dots). The proportion of fusion positive cells versus total number of cells is shown (y axis) versus time, in min (x axis). (C) HIV-1/Vpr-β-Lactamase virions pseudotyped with VSVG turned out not to be fusogenic (red dots) showing the same behavior as HIV1/Vpr-β-Lactamase bald particles (without Env, black dots). (D) HIV-1 virions packaging the Vpr-β-Lactamase chimera and pseudotyped with either VSV-G (cyan dots) or JR-FL (orange dots) were exposed to TZM-bl cells with different concentrations of dynasore (0, 100, 180, 260, 340, and 400 μM) and endpoint BlaM (as defined in Experimental Procedures) was applied. Higher concentrations of dynasore were required to fully inhibit HIV_JRFL_ (240 μM) as compared with HIV_VSVG_ (180 μM). (E) Time-of-addition BlaM kinetics without spinoculation protocols on HIV_VSV-G_ virions using three different blocks: 400 μM dynasore (open red dots), temperature block (pink crosses), and NH_4_Cl (open green dots). All of the kinetics turned out to be very similar. The normalized proportion of fusion positive cells versus total number of cells is shown (y axis) versus time, in min (x axis). (F) Time-of-addition BlaM kinetics without spinoculation protocols on HIV_JRFL_ virions using four different blocks: TAK 779 (open blue dots), dynasore (open red dots), T20 (open black dots), and temperature block (pink crosses). The normalized proportion of fusion positive cells versus total number of cells is shown (y axis) versus time, in min (x axis). In all cases, the error bars represent the SD calculated from three independent experiments.

We tested different concentrations of dynasore assessing HIV_HXB2_ fusion in resting CD4 T cells employing the real-time beta-lactamase assay (BlaM) (Jones and Padilla-Parra, 2016) that measures viral fusion. Briefly, a virion packaging Vpr-BlaM is liberated into the cytoplasm of a target cell and then a Förster resonance energy transfer (FRET) substrate (CCF2) is cleaved and the fluorescence profile altered (Figure 1A). The range of concentrations used in our titration experiments (5, 20, and 80 μM) (Figure 1B) did not affect cell viability, as we detected no propidium iodide (PI) positive cells (Figure S1) under these conditions. Previous reports have shown that the HIV envelope (in this case HXB2), but not the VSV-G protein is capable of mediating HIV infection of resting CD4 T cells (Agosto et al., 2009). Here, we also show that the VSV-G Env was unable to mediate endosomal fusion (Figure 1C) in resting CD4 T cells.

Willing to take a validated model for DNM2-dependent virion endocytosis and fusion, we employed TZM-bl reporter cells previously reported to allow endosomal fusion (Jones and Padilla-Parra, 2016, Miyauchi et al., 2009a, Miyauchi et al., 2009b). HIV_VSV-G_ was able to fuse in TZM-bl cells and is a well characterized virion that fuses within endosomes and is pH dependent (Johannsdottir et al., 2009). Using an endpoint BlaM assay (Zlokarnik et al., 1998), we monitored and compared the impact of different concentrations of dynasore in fusion for both HIV_VSV-G_ and HIV_JRFL_ in TZM-bl cells. Higher concentrations of dynasore were required to fully inhibit fusion for HIV_JRFL_ (250 μM) as compared to HIV_VSV-G_ (180 μM) (Figure 1D), suggesting that the role of DNM2 in HIV_JRFL_ fusion may be unrelated to endocytosis also in TZM-bl reporter cells. Of note, we performed several experiments to validate the use of high concentrations of dynasore on live cells (Figure S1), as it was shown that dynamin inhibitors might have off target effects related to membrane ruffling (Park et al., 2013). We therefore quantitatively studied the impact of dynasore on ruffling and the actin cytoskeleton through the use of FRET Raichu biosensors (Figure S1). We also avoided spinoculation, as we found that this technique might disrupt the regulation of the actin cytoskeleton, as evidenced by changes in small GTPase activity (Figure S1) with a likely knockon effect on endocytosis (Ferguson and De Camilli, 2012). We found that higher dynasore concentrations (250 μM) were needed to arrest full HIV-1 fusion as compared to others (Miyauchi et al., 2009a, de la Vega et al., 2011) (80 μM and 160 μM, respectively). As stressed by de la Vega et al. (2011), it is possible that the dynasore preparation might affect the rate of scape of HIV-1, although dynasore treatment reproducibility blocked HIV-1 endocytosis and fusion in their experiments and ours. This is the reason why we have titrated dynasore (and all drugs employed in our study) while performing cell-viability experiments.

To better understand the role of DNM2 in HIV-1 entry, we performed time-of-addition BlaM (Jones and Padilla-Parra, 2016) using either HIV_VSV-G_ or HIV_JRFL_ in reporter TZM-bl cells. We compared the effect of dynasore with known fusion inhibitors known to disrupt surface accessible viruses (TAK 779 and T20) and universal inhibitors to block fusion for both virions HIV_VSV-G_ or HIV_JRFL_, NH_4_Cl, and temperature block, respectively (Miyauchi et al., 2009a). When treating TZM-bl cells with HIV_VSV-G_ using fully inhibitory concentrations of dynasore (i.e., 400 μM), temperature block, and 80 mM NH_4_Cl, a lysosmotropic agent that raises the endosomal pH and therefore inhibits fusion, we found similar fusion kinetics with t_1/2_ ∼30 min (Figure 1E). This result suggests that, as expected, HIV_VSV-G_ enters the cell via dynamin-dependent endocytosis and the universal inhibitors (NH_4_Cl and temperature block) behave similarly to the specific inhibitor dynasore. Therefore, HIV_VSV-G_ fusion can be completely blocked by inhibiting endocytic pathways. Different fusion inhibitors (point/specific and universal inhibitors) were also utilized when assessing the role of DNM2 in HIV_JRFL_ entry kinetics on TZM-bl cells (Figure 1F). We titrated TAK 779, a small-molecule CCR5 antagonist (Figure S1), and Enfuvirtide (T20), a known fusion inhibitor that blocks the formation of the 6-helix bundle formation (Figure S1), in order to use fully inhibitory concentrations for our time-of-addition BlaM. When plotting together the HIV_JRFL_ fusion kinetics for dynasore, TAK 779, T20, as well as experiments where fusion was inhibited by temperature block (reduction from 37°C to 4°C; Figure 1E), we saw that similar fusion kinetics were obtained for dynasore and TAK779 (specific inhibitors) with similar t_1/2_ = 30 min. Fusion kinetics recovered for T20 and temperature block (universal inhibitors) were also very similar, but both delayed ∼20 min relative to dynasore and TAK77 with t_1/2_ = 50 min. We reasoned that dynasore and TAK 779 must act just prior to fusion, while T20 and the temperature block (universal inhibitor of both endocytosis and fusion) occur right at the moment of fusion pore formation. This result suggests a different role for DNM2 for HIV_JRFL_ as opposed to HIV_VSV-G_. as DNM2 seems to act right before full fusion, almost synchronously with HIV Env/ CD4-CCR5 interaction. Of note, we also tested NH_4_Cl inhibition on HIVJRFL, but as expected it was not able to arrest fusion (Figure S1).

### DNM2 Interactions Are Different for HIV_VSV-G_ and HIV_JRFL_

**Figure 2:**
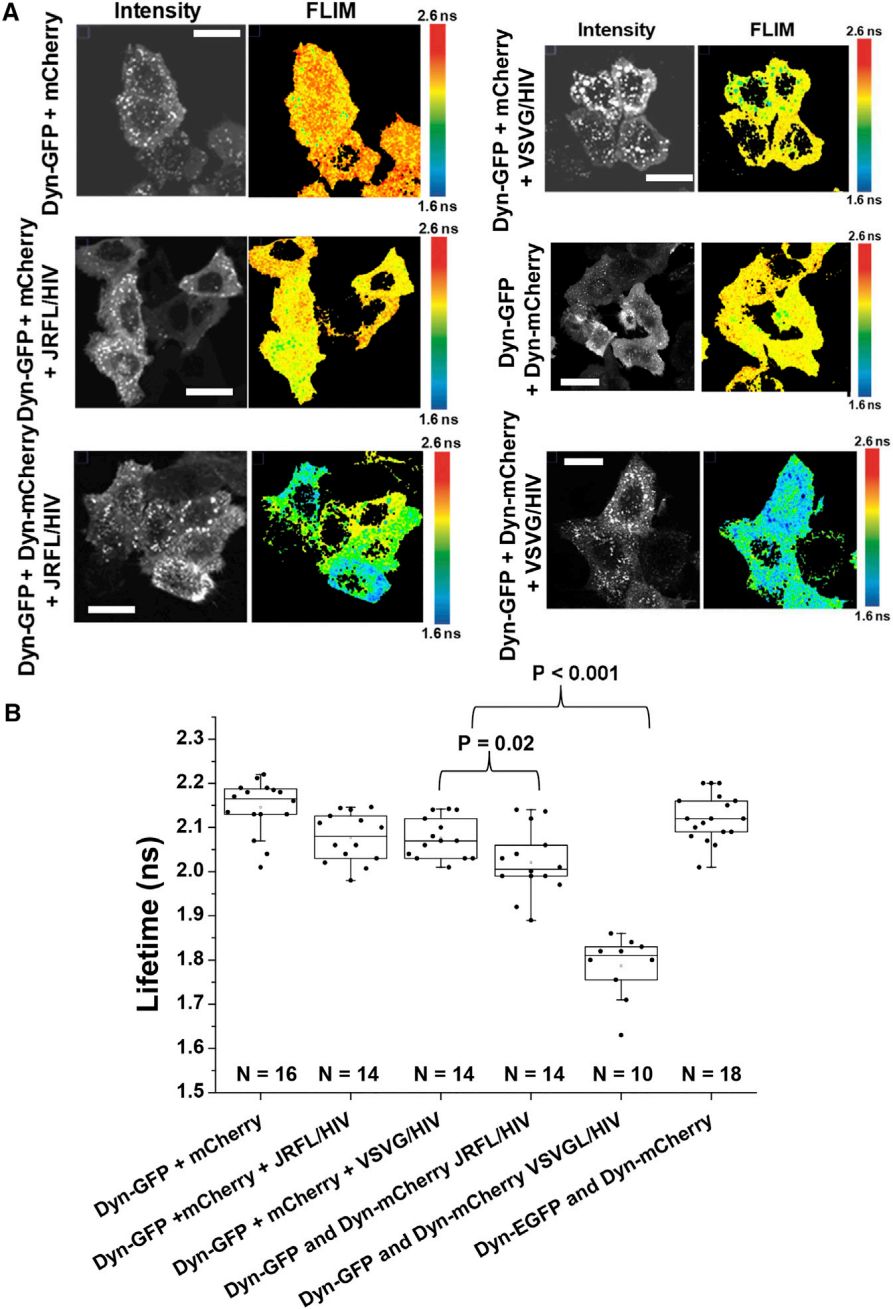
FRET-FLIM Shows Different DNM2 Interactions for HIV_VSV-G_ and HIV_JRFL_ (A) Representative time correlated single photon counting intensity and FLIM micrographs for six different conditions are shown. FLIM images are pseudocolored and blue-cold pixels represent low lifetimes (FRET +), while red-warm pixels represent high lifetimes (FRET −). The negative controls (Dyn-GFP + mCherry with and without virions and Dyn-GFP + Dyn-mCherry) present high average lifetime values (FRET −), while the TZM-bl expressing Dyn-GFP + Dyn-mCherry and exposed to either HIV_JRFL_ or HIV_VSVG_ present blue-colder colors designating FRET+ detection and therefore DNM2 increased interactions upon virus addition. The scale bar represents 15 μm. (B) Boxchart representing the average mean lifetime (in nanoseconds, ns) recovered from individual cells from at least three different FRET-FLIM experiments (n = 3) is shown for different conditions. The conditions are as follows: TZM-bl cells expressing Dyn-GFP + mCherry diffusing alone (n = 16), TZM-bl cells expressing Dyn-GFP + mCherry diffusing alone in the presence of HIV_JRFL_ (n = 14), TZM-bl cells expressing Dyn-GFP + mCherry diffusing alone in the presence of HIV_VSVG_ (n = 14), TZM-bl cells expressing Dyn-GFP + Dyn-mCherry in the presence of HIV_JRFL_ (n = 14), TZM-bl cells expressing Dyn-GFP + Dyn-mCherry in the presence of HIV_VSVG_ (n = 10), and TZM-bl cells expressing Dyn-GFP + Dyn-mCherry (n = 18).

Recently, a report showed the importance of using FLIM to follow DNM2 activity in live cells in relation with its role regulating actin dynamics (Gu et al., 2014). We therefore applied FLIM to follow DNM2 interactions in live cells in the context of virus entry and fusion (Figures 2A and 2B). TZM-bl cells co-transfected with DNM2 labeled with either eGFP (Dyn-GFP) or mCherry (Dyn-mCherry) were exposed to HIV_JRFL_ or HIV_VSV-G_ at high MOIs (10). As a negative control, viruses at the same MOI were also added to TZM-bl cells co-transfected with Dyn-GFP and mCherry alone. A shortening of the average lifetime due to FRET was observed for TZM-bl cells co-transfected with Dyn-GFP and Dyn-mCherry being treated with HIV_JRFL_ or HIV_VSV-G_ (p = 0.02 and p < 0.001, respectively), indicating DNM2 interacting (Gu et al., 2014). Importantly, HIV_VSV-G_ exposure resulted in a drastic lifetime diminution (average < τ > = 1.78 ± 0.07 ns, n = 10 as compared to the control < τ > = 2.16 ± 0.09 ns, n = 18), whereas HIV_JRFL_ exposure produced only a slight, but significant lifetime diminution (average < τ > = 2.02 ± 0.07 ns, n = 14) when compared to the negative controls. These data suggest that the VSV-G envelope protein—and to a lesser extent that of JRFL—provoked DNM2 to interact, albeit to different extents (Figure 2B). It is therefore possible that DNM2 plays distinct roles in the entry mechanisms of HIV_VSV_ and HIV_JRFL_. Of note, the distribution of endocytic markers (early and mature endosomes, Rab5-mCherry) did not change upon addition of HIV virions (Figure S2).

### HIV-1 Entry and Fusion Require a DNM2 Low Oligomeric State

**Figure 3:**
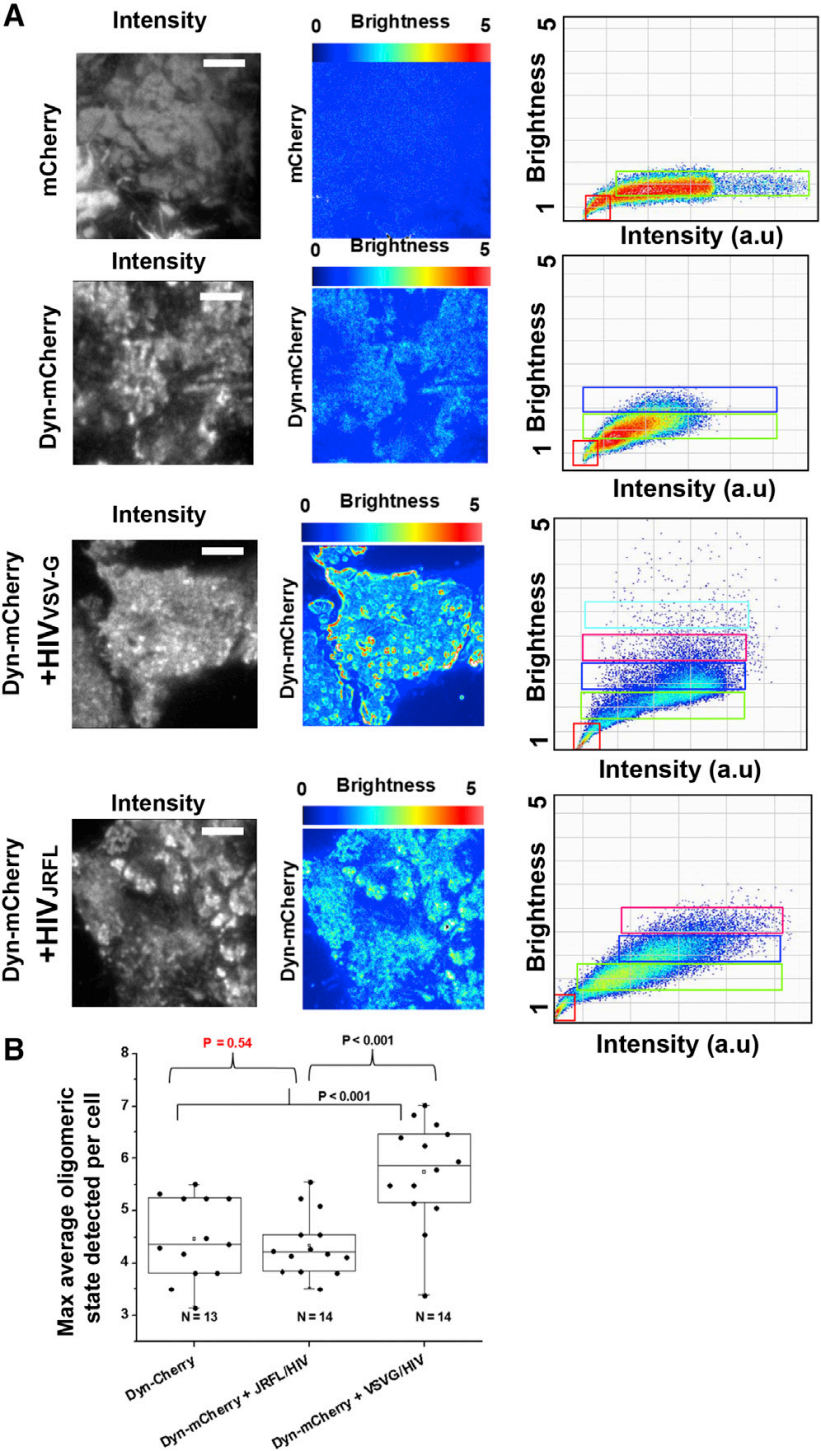
Live Cell TIRF-Number and Brightness Analysis Shows Low Oligomeric States for Dynamin-2 when Cells Are Exposed to Virions with HIV Env (A) TZM-bl cells expressing mCherry alone (first row), Dynamin-mCherry (second row), Dynamin-mCherry treated with HIV_VSVG_ (third row), and Dynamin-mCherry treated with HIV_JRFL_ (fourth row) were imaged using TIRF (as described in Experimental Procedures). The average intensity images (first column from the left, gray micrographs) are shown together with the brightness images (second column from the left, rainbow pseudocolor), and the graph plotting brightness (counts per second per molecule) versus intensity (arbitrary units) for all pixels is also shown (third column from the left). The high oligomeric states are seen in cells treated with HIV_VSVG_ (red pixels with high brightness, warm colors), and the lower oligomeric states comparable to Dynamin-mCherry without treatment were seen in cells exposed to HIV_JRFL._ In both cases, the cells were treated with MOI = 10. The size of the micrographs is 25.6 × 25.6 μm. (B) The average maximum oligomeric state detected per cell is plotted for three different conditions: TZM-bl cells expressing Dynamin-mCherry (first column), TZM-bl cells expressing Dynamin-mCherry treated with HIV_JRFL_ (second column), and TZM-bl cells expressing Dynamin-mCherry treated with HIV_VSVG_. The higher oligomeric states where detected taking as a reference the brightness recovered from mCherry alone (monomers) expressed in TZM-bl cells and calibrating the S factor of the EM-CCD camera as explained in Experimental Procedures. Only cells treated with HIV_VSVG_ systematically showed higher oligomeric states right after addition of the virions, indicating high CME endocytic activity.

In a previous report (Ross et al., 2011), the oligomeric state of DNM2 close to the plasma membrane was investigated by combining TIRFM with number and brightness analysis (Unruh and Gratton, 2008). When combining TIRF with number and brightness utilizing a very fast image acquisition (i.e., 50 ms/frame), it is possible to quantify the oligomeric state of Dynamin if the dwell time (image acquisition) is less than that of the diffusion being investigated. Number and brightness analysis provides quantitative information regarding the oligomeric state on a pixel by pixel basis. Since we had seen changes in lifetime that relate with protein-protein interactions of DNM2 in the previous FRET-FLIM experiment, we further investigated this finding by expressing Dyn-mCherry in TZM-bl cells before exposing them to either HIV_JRFL_ or HIV_VSV-G_ and performing TIRF/number and brightness microscopy (Figure 3). We also show that virions are able to get underneath the cells using labeled virions (HIV_JRFL_ Gag-GFP) and TZM-bl cells expressing Dyn-mCherry (Figure S3). The addition of HIV_VSV-G_ at a MOI of ten induced the formation of higher oligomeric states (octamers, red pixels in the N and B figures, Figure 3), suggesting that the scission of CCPs during CME may be conducted by dynamin octamers (n = 14). Conversely, the addition of HIV_JRFL_ at the same MOI had no noticeable effect on the oligomeric state of Dyn-mCherry (tetramers, identified in Figure 3, n = 14). Thus, HIV entry appears not to require higher-order Dynamin structures in TZM-bl cells.

### Dynamin-2 Stabilizes the Fusion Pore during HIV Fusion

**Figure 4:**
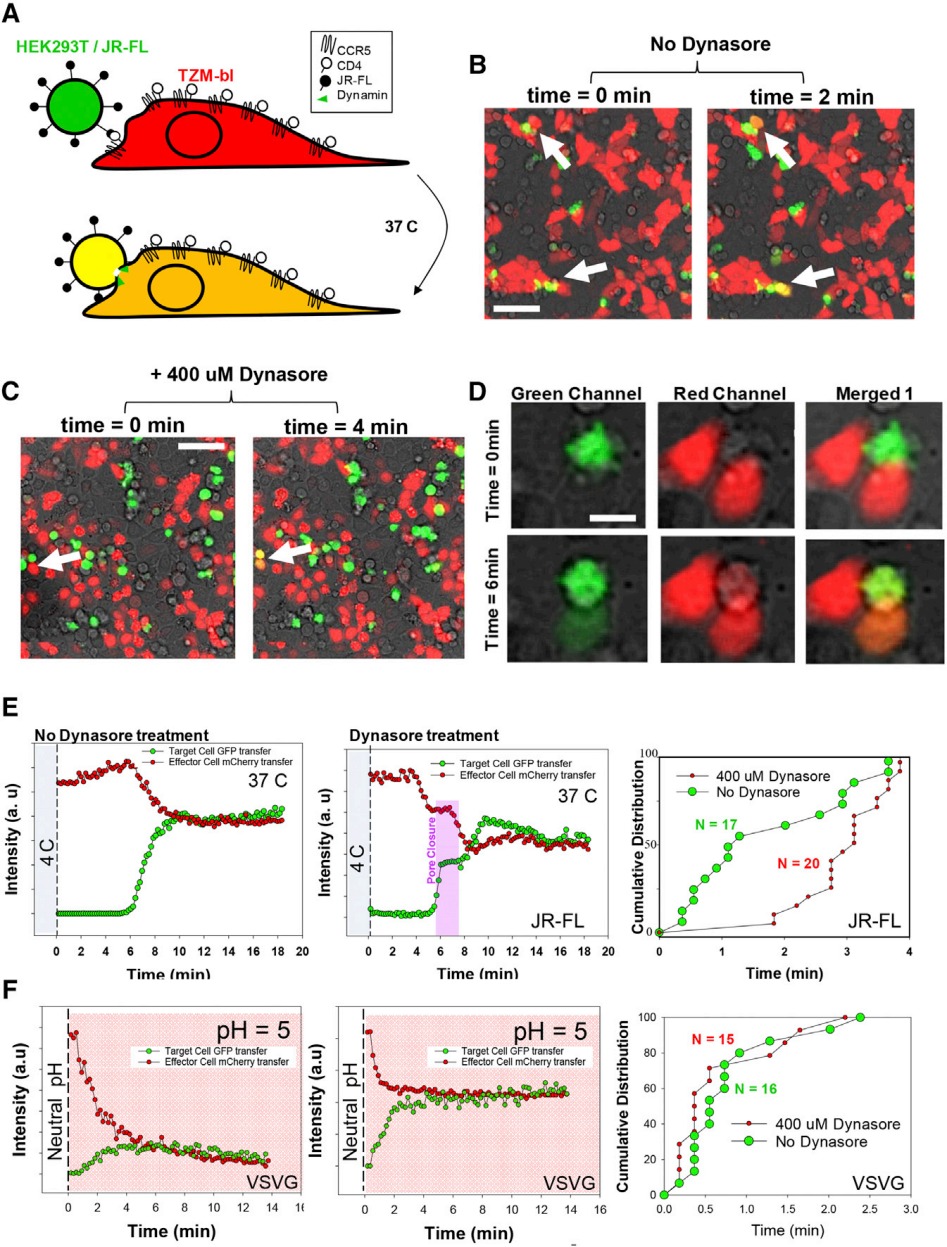
Cell-Cell Fusion Experiments Show that Dynamin-2 Stabilizes HIV-1 Env Mediated Fusion (A) A cartoon depicting the strategy followed for our cell-cell fusion assays is shown. Briefly, HEK293T cells expressing freely diffusing GFPs and JRFL Env (effector cells) were added onto TZM-bl reporter cells expressing freely diffusing mCherry (target cells) at 4°C for 30 min. Shifting the temperature under the microscope at 37°C permitted the visualization of JRFL Env mediated cell-cell fusion, measured by time-resolved two color confocal fluorescence microscopy. (B and C) Micrographs showing TZM-bl cells transfected with mCherry exposed to HEK293T cells expressing JR-FL and eGFP untreated (B) or treated with 400 μM of dynasore (C). The transmission channel is also included (in gray). Different time points show cells undergoing cell-cell fusion 2 min after changing the temperature to 37°C (B) or 4 min (C). These events are shown with a white arrow. The error bar represents 20 μm. (D) Composite micrographs of a region of interest depicting cell-cell fusion showing transmission and green channel (HEK293T cells expressing GFP and JRFL Env, left column), transmission and red channel (TZM-bl cells expressing mCherry, middle), and merged channels (right column) at two different time lags: 0 min (no fusion) and 6 min (cell-cell fusion completed shown by the concomitant transfer of red mCherry fluorescent proteins from target cells toward effector cells and eGFP fluorescent proteins from effector cells toward target cells). The scale bar represents 10 μm. (E) The fluorescence intensities were recovered as a function of time integrating both signals (red and green) coming from two single events from target cells in the absence of dynasore (left) and in the presence of 400 μM dynasore (middle). The flickering of the fusion pore is only observed in cells treated with dynasore. The cumulative distribution of individual cell-cell fusion events comparing untreated cells (green dots, n = 17) against dynasore treated cells (small red dots, n = 20) is shown in the right image, evidencing a delay of around 3 min for cells treated with DNM2 inhibitor dynasore. (F) HEK293T cells expressing freely diffusing GFPs and VSVG Env (effector cells) were added onto TZM-bl reporter cells expressing freely diffusing mCherry (target cells) at room temperature for 30 min. Shifting the pH using a citrate buffer at pH ∼5 permitted us to visualize VSVG Env mediated cell-cell fusion, measured by time-resolved two color confocal fluorescence microscopy. The left image shows a representative example without dynasore treatment, and the middle image being an example of cell-cell fusion treated with 400 μM dynasore. Flickering of the pore was never observed in this case. The cumulative distribution of individual cell-cell fusion events comparing untreated cells (green dots, n = 16) against dynasore treated cells (small red dots, n = 15) is shown in the right image, evidencing synchronous fusion kinetics.

Fusion between individual HEK293T effector cells expressing the JRFL envelope and cytosolic eGFP and target TZM-bl reporter cells expressing mCherry was studied using real-time fluorescence microscopy (Figure 4). Effector HEK293T cells were allowed to sediment on target cells at 4°C for 30 min (as described in Experimental Procedures), sufficient time to allow receptor priming (Padilla-Parra et al., 2013). Subsequently, the sample was mounted on an inverted microscope and the temperature shifted to 37°C in order to allow cell-cell fusion to occur (Figure 4A). The formation of fusion pores and the kinetics of fusion were assessed by the transfer of eGFP from the effector cells toward the target cells that mirrored the mCherry transfer of target cells toward effector cells (Figures 4B–4D). Both effector and target cells became yellow when equilibrium in fusion pore dynamics was established (Figures 4B–4D). Changes in the mean fluorescence intensity of the target (red signal) and the effector cells (green signal) were plotted (Figure 4E, left). When cells were treated with high concentrations of Dynasore (400 μM) flickering of the pore was observed (Figure 4E, middle), indicating that the fusion pore was not stable under these conditions (Padilla-Parra et al., 2012). There is a slight possibility that the pore closure, measured as stabilization of the GFP and concomitant mCherry transfer from effector and target cells (pink zone in Figure 4E, middle), comes from several pores simultaneously, but they should have to be totally synchronized, as opening and closure at different times would never be able to arrest fusion over 2 min (horizontal lines for time-dependent intensities of GFP and mCherry in the pink zone for flickering).

In all cases, delayed pore formation and pore closing was observed for TZM-bl cells treated with dynasore, suggesting that DNM2 plays an important role in establishing and stabilizing the HIV-1 fusion pore. When plotting the cumulative distribution of individual fusion events coming from three independent experiments, a delay in ∼3 min was observed for the TZM-bl cells treated with dynasore relative to the untreated ones (Figure 4E, right). The average t_1/2_ for cell-cell (JR-FL) fusion events without dynasore treatment was 2.83 ± 1.69 min (n = 17); the average cell-cell fusion event for dynasore treated cells was delayed when taking into consideration the initial (pore opening) and final points (equilibrium) 4.9 ± 1.8 min (n = 20). Inhibitory concentrations for single virus fusion in the presence of T20 or TAK 779 (Ayouba et al., 2008), known HIV-1 fusion inhibitors that block fusion and receptor engagement, respectively, were able to arrest fusion (Figure S4), providing a robust negative control for the cell-cell fusion approach. In contrast, when cell-cell fusion was studied using effector HEK293T cells expressing the VSV-G envelope and TZM-bl cells expressing mCherry, no change in pore formation or kinetics of individual events was observed when dynasore was present (Figure 4F). Cell-cell fusion constitutes a unique approach to study fusion in the absence of endocytosis and showed that DNM2 is needed to establish and maintain the fusion pore (Figure S4) only in HIV-1 and not VSV, suggesting that there must be a regulation step in HIV-DNM2 dependent fusion; as DNM2 is recruited toward the fusion pore perhaps through HIV-1/CD4 and co-receptor interactions.

### Dynamin-2 Co-localizes with Double Labeled HIV-1 Virions prior to Fusion

**Figure 5:**
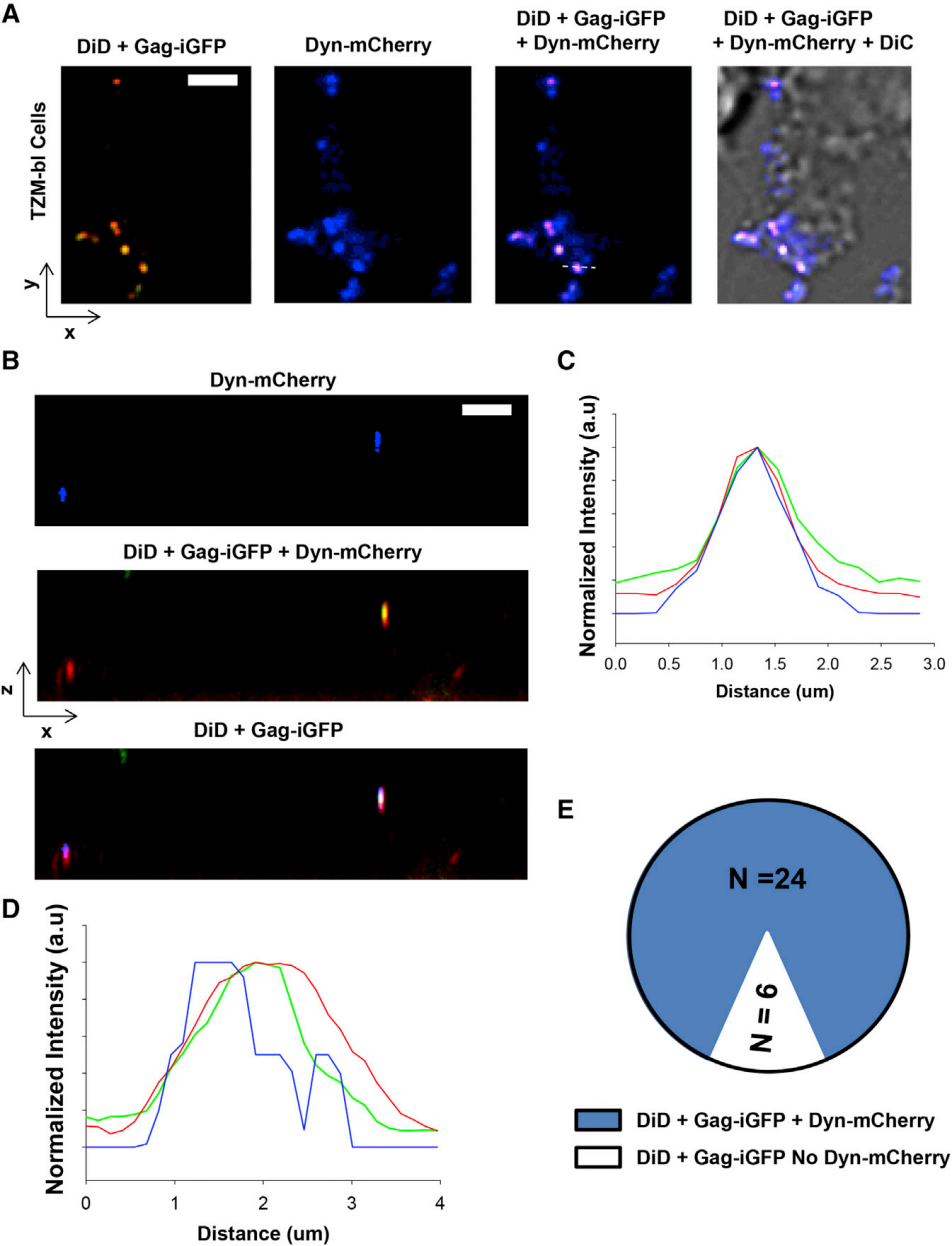
HIV_JRFL_ Virions Co-localize with Dynamin prior to Fusion (A and B) Confocal imaging of TZM-bl cells expressing DNM2-mCherry (in blue) exposed to double labeled HIV_JRFL_ virions (DiD/Gag-GFP, yellow) were imaged right after receptor priming in X-Y (A) and X-Z (B) directions. The scale bars represent 0.5 μm. (C and D) The line histograms for two representative particles in X-Y (C) and X-Z (D) are shown. In both cases the integrals under the DNM2-mCherry curves overlapped >80% as compared to the integrals coming from Gag-GFP and DiD indicating positive co-localization. (E) Statistics from at least three independent experiments showing that 80% of the particles analyzed (24 out of 30) showed positive co-localization between the virions and DNM2-mCherry prior fusion.

In order to test whether DNM2 is recruited in the inner plasma membrane toward primed HIV_JRFL_ virions prior fusion, we imaged TZM-bl cells expressing DNM2-mCherry in the presence of double labeled virions HIV_JRFL_ (DiD/Gag-GFP). The virions were allowed to prime CD4 receptors in TZM-bl cells for 30 min at 4°C. Again, spinoculation protocols were not applied to avoid unwanted side effects in DNM2 regulation. The cells were imaged under the microscope and micrographs acquired both in X-Y and X-Z directions in a confocal microscope as explained in Experimental Procedures. Co-localization analysis in both planes revealed that 75% of the double labeled particles analyzed co-localized with DNM2 before fusion (Figure 5), as both the envelope (DiD labeled) and the core (GFP-Gag) co-localized with DNM-2-mCherry. We examined the spatial overlap between the intensity profiles for DNM2-mCherry and DiD/Gag-GFP that was above 80% in all cases positive for co-localization in both directions X-Y and X-Z, n = 24 from three independent experiments (Figures 5C and 5D). These results suggest that DNM2 recruitment happens prior to fusion. We also tested the dominant-negative mutant DNM2-K44A in the context of HIV-1 fusion and found that it was not able to fully block HIV_JRFL_ fusion (Figure S5). These data coincide with Herold et al. (2014) and supports the idea of DNM2 having a low oligomeric state as DNM2 GTPase activity relates with high oligomeric states (Ferguson and De Camilli, 2012).

## Discussion

**Figure 6:**
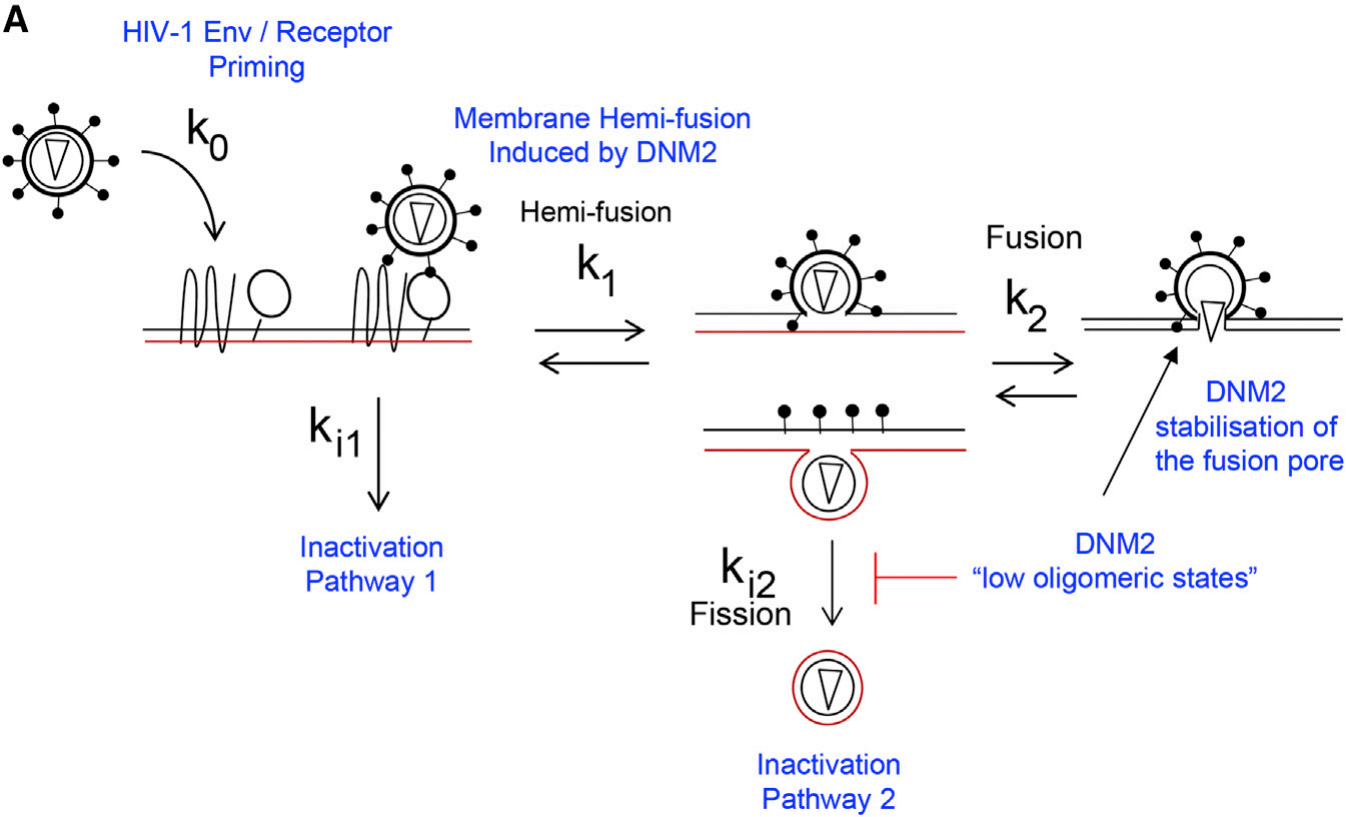
Cartoon Depicting the Model Proposed for HIV-1 Entry and Fusion and the Role of DNM2 in this Process (A) Cartoon summarizing the potential role of DNM2 during HIV-1 entry and fusion. Right at the moment of HIV-Env priming with the co-receptor membrane HIV-1 hemi-fusion might occur. Low oligomeric states of DNM2 potentially block fission and also help stabilize the fusion pore. The black line represents the outer lipid leaflet and the red line the inner leaflet.

The mode of entry for HIV-1 was thoroughly investigated in a recent report (Herold et al., 2014), where the authors determined that HIV-1 must fuse in the plasma membrane and that HIV-1 does not require endocytosis to complete fusion. This view, however, is opposed to that of Miyauchi et al. (2009a), who postulated that HIV-1 has to undergo exclusively endosomal fusion based on data from real-time single virus tracking combined with BlaM assays. We suspect that this controversy in the field debating whether or not HIV-1 gets inside the cell through endocytosis (Marin and Melikyan, 2015) has deviated the attention from the actual role of DNM2 during HIV-1 fusion. Nevertheless, there is growing interest in the field to understand the role of actin dynamics in HIV infection: a recent report (Ménager and Littman, 2016) points at the importance of DNM2 in dendritic cells mediated trans-enhancement of CD4 T cell infection by HIV in vitro. In this scenario, insights about the true role of DNM2 during single virus fusion are needed to fully understand the mechanisms taking place (Padilla-Parra and Dustin, 2016). Indeed, the process of HIV-1 fusion pore formation and enlargement is an energy-intensive mechanism that necessitates the orchestrated role of several proteins (Munro et al., 2014), among them DNM2. Membrane fusion is vital for eukaryotic live, in this context it has recently been shown the transition to full membrane fusion can be determined by competition between fusion and DNM2-dependent fission mechanisms supporting the hemi-fusion and hemi-fission hypothesis in live cells (Zhao et al., 2016). Our data suggest that DNM2 might play a multifaceted role during HIV-1 entry: first, a low DNM2 oligomeric state (n = 4) might help to induce HIV-1 hemi-fusion (Montessuit et al., 2010) and in turn prevent fission from happening as DNM2 fission depends on the formation of an octamer with a ring like structure and GTPase activity (Mattila et al., 2015). These sequences of events would favor HIV-1 full fusion and second, DNM2 tetramers could concomitantly stabilize the fusion pore right after HIV-1 hemi-fusion (Figure 6). Here, we show various lines of evidence to support this hypothesis: first, we have shown substantial changes to HIV-1 fusion kinetics when primary CD4 T cells are treated with a low dosage (non-inhibitory concentration) of dynasore (5 μM and 20 μM) (Figure 1). We have also shown that dynasore acts right before fusion synchronously with TAK 779, a CCR5 antagonist (Figure 1) in reporter TZM-bl cells. Second, our quantitative imaging experiments based on FRET-FLIM (Figure 2) and number and brightness (Figure 3) clearly show a difference in DNM2 activity and oligomeric state when treating the cells with high concentration of either HIV_VSVG_ (high oligomers, octamers) or HIV_JRFL_ (low oligomers, tetramers). Third, cell-cell fusion assays revealed that dynasore could disrupt the formation of the fusion pore between effector cells expressing the HIV-1 Env (JRFL) and target TZM-bl cells, causing flickering of the fusion pore and delayed fusion kinetics (Figure 4). However, we could not fully inhibit fusion with high concentrations of dynasore. Importantly, the dominant-negative mutant DNM2-K44A was not able to fully block HIV_JRFL_ fusion (Figure S5). This mutant blocks DNM2 GTPase activity that in turn is related to its oligomeric state (Ferguson and De Camilli, 2012), reinforcing the idea that DNM2 acts with a low oligomeric state during HIV-1 entry and fusion and also that its role during this process is not related to endocytosis. Moreover, we also show that DNM2 recruitment toward the fusion pore has to be specific (Figure 5) and regulated, and this suggests that it might be responsible to induce HIV-1 hemi-fusion as a tetramer. This behavior has previously been reported for a Dynamin related protein 1 (Drp1) that promotes tethering and hemi-fusion of membranes in vivo (Montessuit et al., 2010). This DNM2 tetrameric state in turn would be very important since on one hand it is unable to complete fission (Ferguson and De Camilli, 2012) and on the other it induces full fusion and pore stabilization (Figure 5). We hypothesize that DNM2 might be regulated by engagement of CD4 and co-receptor interactions either through a retroactive loop with actin as suggested in Taylor et al. (2012) and/or through a BAR domain protein able to sense curvature (González-Jamett et al., 2013). Overall our data suggest that DNM2, as a tetramer, might help to establish hemi-fusion, might inhibit fission, and does stabilize the pore during HIV-1 fusion.

## Experimental Procedures

### Plasmids

pR8ΔEnv (encoding the HIV-1 genome harboring a deletion within Env), pcRev, Gag-GFP, H1N1, and VSV-G were kindly provided by Greg Melikyan (Emory University). The plasmid encoding the JR-FL envelope protein was a kind gift from James Binley (Torrey Pines Institute for Molecular Studies). Dynamin-EGFP and Dynamin-mCherry where obtained from Addgene.

### Cell Culture

HEK293T cells and TZM-bl cells were grown using DMEM (Life Technologies) supplemented with 10% fetal bovine serum, 1% penicillin-streptomycin, and 1% L-Glutamine to give DMEM complete (DMEM_comp_). All cells were maintained in a 37°C incubator supplied with 5% CO_2_.

### Cell Purification

Leukoreduction chambers from healthy individuals were obtained from the National Blood Service. CD4+ T cells were purified from the peripheral blood of healthy human donors. Blood was incubated (20 min, 25°C) with RosetteSep human CD4+T cell enrichment cocktail (StemCell Technologies). The remaining unsedimented cells were loaded onto Ficoll-Paque Plus (Sigma-Aldrich), isolated by density centrifugation, and washed with PBS. The purified cells were cultured in RPMI containing antibiotics and 10% heat-inactivated FBS. De-identified leukoreduction chambers were obtained from the Oxford Radcliffe Biobank, which operates under UK Human Tissue Authority license number 12217.

### Flow Cytometry

Purified CD4+ T cells were stained (30 min, 4°C) with APC-labeled anti-CXCR4 (eBioscience) and FITC-labeled anti-CD4 (eBioscience) antibodies and washed with PBS (containing 0.05% BSA and 0.05% sodium azide). We analyzed samples in a LSR II machine (BD) and FlowJo software.

### Virus Production

Pseudotyped viral particles were produced by transfecting HEK293T cells plated at ∼60%–70% confluency in T75 or T175 flasks. DNA components were transfected using GeneJuice (Novagen) in accordance with the manufacturer’s instructions. To produce particles harboring the BlaM protease, cells were transfected with 2 μg pR8ΔEnv, 2 μg Vpr-BlaM, 1 μg pcREV, and 3 μg of the appropriate viral envelope (either VSV-G or the CCR5-tropic HIV-1 strain JR-FL or the CXCR4-tropic HXB2). For viruses harboring Gag-GFP, 3 μg of the Gag-GFP plasmid were used. Transfection mixtures were then added to cells in DMEM_comp_ before returning flasks to the 37°C CO_2_ incubator. At 12 hr post-transfection, the transfection mixture-containing medium was removed and cells were washed with PBS. Fresh DMEM_comp_ (lacking phenol red) was then added. Cells were subsequently incubated for a further 24 hr. At 48 hr post-transfection, viral supernatants were removed from cells and pushed through a 0.45 μm syringe filter (Sartorius Stedim Biotech) before being aliquoted and stored at −80°C. For SVT-compatible virus production, cells were transfected in the same manner with 2 μg pR8ΔEnv, 3 μg Gag-GFP, 1 μg pcREV, and 3 μg of the appropriate viral envelope (either VSV-G or JR-FL). At 12 hr post-transfection, the transfection complexes were removed and cells were washed with PBS before being incubated at 37°C with 10 mL Opti-MEM (Life Technologies) containing 10 μM DiD (Life Technologies) for 4 hr. Subsequently, the staining mixture was removed, cells washed twice with PBS, and fresh DMEM_comp_ (lacking phenol red) was then added. Cells were incubated for a further 24 hr prior to harvesting.

### BlaM Assay

At 24 hr prior to the assay, TZM-bl cells were plated at 4 × 10^4^ cells/well in black clear-bottomed 96 well plates. On the day of assay, cells were cooled on ice prior to the addition of the appropriate MOI of virus (all infections were performed in 100 μL volumes). Immediately following addition of virus harboring Vpr-BlaM, cells were placed at 4°C for 1 hr. Virus was then removed and cells were washed with PBS and 100 μL of DMEM_comp_ was added to each well before shifting the plate to the 37°C CO_2_ incubator to initiate viral entry. To gain kinetic data, virus fusion was blocked at the appropriate time point (0, 15, 30, 45, 60, 75, and 90 mins) by removing the media and replacing with media containing Dynasore, TAK 779, NH_4_Cl, or T20 (Sigma-Aldrich). The inhibitor concentrations were found by testing different concentrations in titration experiments (Supplemental Information). Note that for the 0 min time point, drugs were added immediately prior to the 37°C temperature shift. After 90 mins, cells were loaded with CCF2-AM from the LiveBLAzer FRET B/G Loading Kit (Life Technologies) and incubated at room temperature in the dark for 2 hr. Finally, the CCF2 was removed; cells were washed with PBS and fixed with 2% PFA prior to viewing.

### BlaM Assay Spectral Analysis and Real-Time BlaM

TZM-bl cells loaded with CCF2 were excited using a 405 nm continuous laser (Leica) and the emission spectra between 430–560 nm was recorded pixel by pixel (512 × 512) using a Leica SP8 X-SMD microscope with a lambda resolution of 12 nm. The ratio of blue emission (440–480 nm, cleaved CCF2) to green (500–540 nm, uncleaved CCF2) was then calculated pixel by pixel using ImageJ (https://imagej.nih.gov/ij/) for three different observation fields using a 20× objective and plotted as a function of time. Fusion kinetics were then recovered with automated software (R) detecting blue/green ratios coming from individual cells above the threshold given by our negative control (No Env virions packaging Vpr-BlaM).

Finally, a new protocol able to retrieve real-time HIV-1 fusion data was applied. Briefly, the real-time-BlaM assay represents a more streamlined approach for measuring virus fusion kinetics. Here, target cells are first loaded with the CCF2-AM in the presence of 12.5 mM probenecid and later exposed to virus particles. This means upon temperature shift to 37°C, cleavage of CCF2-AM and the resultant color change from green to blue can be visualized in real time, all in a single sample of cells/virus and without the need for fusion inhibitor addition. This typically permits the recording of more data sets and produces a more refined kinetic curve as compared to time-of-addition BlaM. Of note, this protocol was also applied on TZM-bl cells, but without success. We found that the CCF2-AM substrate was pumped out more efficiently in these cells even in the presence of probenecid and therefore decided to apply a time-of-addition approach with TZM-bl cells.

### Förster Energy Transfer by Fluorescence Lifetime Imaging Microscopy

Living cells expressing Dynamin-EGFP alone or co-expressing Dynamin-EGFP and Dynamin-mCherry were imaged before and after virion addition using a SP8–X-SMD Leica microscope from Leica Microsystems. Areas of interest were chosen under either a 20× air immersion objective or a 63×/1.4 NA oil immersion objective. Cells were excited using a 488 nm pulsed laser tuned at 80 MHz coupled with single photon counting electronics (PicoHarp 300) and subsequently detected by hybrid external detectors. To rule out artifacts due to photo-bleaching and insufficient signal to noise, only cells with at least 250–1,000 photons per pixel and negligible amount of bleaching were included in the analysis after a 2 × 2 image binning (Leray et al., 2013, Padilla-Parra et al., 2009). The acquired fluorescence decay of each pixel in one whole cell was deconvoluted with the instrument response function (IRF) and fitted by a Marquandt nonlinear least-square algorithm with one or two-exponential theoretical models using Symphotime software from Picoquant GmbH. The mean fluorescence lifetime (Tau) and fraction of interacting donor (f_D_) were calculated as previously described (Leray et al., 2013, Zhao et al., 2014) using SymPhoTime, Mapi software (Leray et al., 2013) and ImageJ (https://imagej.nih.gov/ij/). Statistical analysis of the lifetime data was performed using a two-tailed t test or rank-sum test (SigmaPlot). A mask to filter out the punctate structures based on threshold analysis was applied using ImageJ showing that the overall average lifetimes did not change. TCSPC acquisitions lasted ∼3 min to accumulate enough photons in order to perform double exponential fits. Importantly, transient interactions or high intensity structures will be exaggerated after accumulating photons during the acquisition times.

### Total Internal Reflection Microscopy Combined with Number and Brightness Analysis

TZM-bl cells were transfected with Dynamin-mCherry and observed in a Zeiss Elyra TIRF microscope equipped with a 100× oil objective (1.46 NA). Cells were exposed to a 561 nm line (100 mW) and total internal reflection was achieved reaching the critical angle (previously calibrated with lipid-bilayers treated red lipophilic dyes). There were 100 images that were recovered at 256 × 256 pixels setting the EM-CDD (Andor) exposure time at 50 ms per frame. Images were analyzed to recover number and brightness using SimFCS software (Laboratory for Fluorescence Dynamics, University of California at Irvine). In order to avoid for cell movement and moving objects, a running average of ten frames was used to detrend the fluorescence fluctuation and correct for cell movement during the acquisition. A sample with cells expressing mCherry alone was used to calibrate the settings of the system and recover a brightness above 1 for molecular diffusion above immobile structures and detector noise.

### Cell-Cell Fusion Assays

HEK293T cells expressing freely diffusing GFPs and JRFL Env (effector cells) were added onto TZM-bl reporter cells expressing freely diffusing mCherry (target cells) at 4°C for 30 min. Shifting the temperature under the microscope at 37°C permitted to visualize JRFL Env mediated cell-cell fusion, measured by time-resolved two color confocal fluorescence microscopy using a Leica SP8 microscope. A white light laser (WLL) was set at 488 and 588 nm to simultaneously excite GFP and mCherry using a 40× oil immersion objective and the emission light of both fluorescent proteins was recovered with photon counting detectors (HyD) tuned at 500–550 (green channel) and 600–650 (red channel). The pinhole was set at 1.5 Airy units, and we used an automatic adaptive autofocus to prevent z-drifting while imaging (Leica). Leukoreduction chambers were used as a source of human peripheral blood mononuclear cells. The fluorescence intensities were recovered as a function of time integrating both signals (red and green) coming from regions of interest comprising target cells (TZM-bl) in the absence of dynasore and in the presence of 400 μM dynasore using ImageJ free software (https://imagej.nih.gov/ij/). If cells moved during the movies, single cell tracks were recovered using manual tracking (ImageJ). The cumulative distribution of individual cell-cell fusion events was calculated using Sigma Plot. The concentration of T20 (Sigma) used to inhibit cell-cell fusion was 40 μg/mL.

Time-resolved single virus tracking with TIRFM was performed on TZM-bl cells expressing DNM2-mCherry (Addgene) that were grown to near confluency on glass-bottom 35 mm Petri dishes (MatTek) in phenol red-free growth medium. Cells were placed at 4°C and HIV_JRFL_ viruses (packaging Gag-GFP) at 1.5 × 10^4^ IU were added and allowed to sediment down for ∼30 min. After that, cells were placed under the TIRF microscope and imaged using a 100× objective using a 488 nm laser for GFP and 561 for mCherry.

### 3D Confocal Imaging

TZM-bl cells expressing either DNM2-mCherry (Addgene) or Rab5-mCherry were grown to near confluency on glass-bottom 35 mm Petri dishes (MatTek) in phenol red-free growth medium. Cells were placed at 4°C and viruses at 1.5 × 10^4^ IU were added and allowed to sediment down for ∼30 min. After that, cells were placed under the SP8XSMD Leica confocal microscope (Leica Microsystems) and imaged. WLL was set for two different pathways to avoid bleed-through between Gag-GFP, Rab5-mCherry, and DNM2-mCherry: (1)WLL tuned at 488 and 633 nm to simultaneously excite GFP and DiD and (2) WLL tuned at 589 to excite DNM2-mCherry. We used a 63× oil immersion (1.3 NA) objective and the emission light of both fluorescent proteins and DiD were recovered with photon counting detectors (HyD) tuned at 500–550 (green channel), 600–650 (mCherry channel channel), and 640–700 (DiD channel). The pinhole was set at 1 Airy unit, and we used an automatic adaptive autofocus to prevent z- and y-drifting while imaging (Leica). Images were taken in X-Y and X-Z planes. The fluorescence intensity profiles were recovered integrating both pathways: (1) signals (DiD, far-red and Gag-GFP, green) and (2) DNM2-mCherry (shown in blue) coming from lines crossing the equatorial part of double labeled virions using ImageJ free software (https://imagej.nih.gov/ij/). Co-localization was considered to be positive when the overlap between the DNM-mCherry intensity profile was at least 80% with both channels DiD and Gag-GFP.

## Author Contributions

S.P.-P. and M.L.D. conceived and designed research; D.M.J., L.A.A., R.N., M.F., R.S.U., X.M.-M., H.N.-K., and S.P.-P. performed research and analyzed the data. S.P.-P. wrote the manuscript with comments from all authors.

## Supplemental Information

Document S1. Supplemental Experimental Procedures and Figures S1–S5

Document S2. Article plus Supplemental Information
