# Supplementary material for: Dynamin-2 Stabilizes the HIV-1 Fusion Pore with a Low Oligomeric State
Source: Cell Rep. 2017 Jan 10;18(2):443–53. doi: 10.1016/j.celrep.2016.12.032 (PMC5263234; doi:10.1016/j.celrep.2016.12.032)
Supplement: Document S1. Supplemental Experimental Procedures and Figures S1–S5 [file mmc1.pdf]

Cell Reports, Volume *18*

## Supplemental Information

# Dynamin-2 Stabilizes the HIV-1 Fusion Pore with a Low Oligomeric State

**Daniel M. Jones, Luis A. Alvarez, Rory Nolan, Margarita Ferriz, Raquel Sainz Urruela, Xènia Massana-Muñoz, Hila Novak-Kotzer, Michael L. Dustin, and Sergi Padilla-Parra**

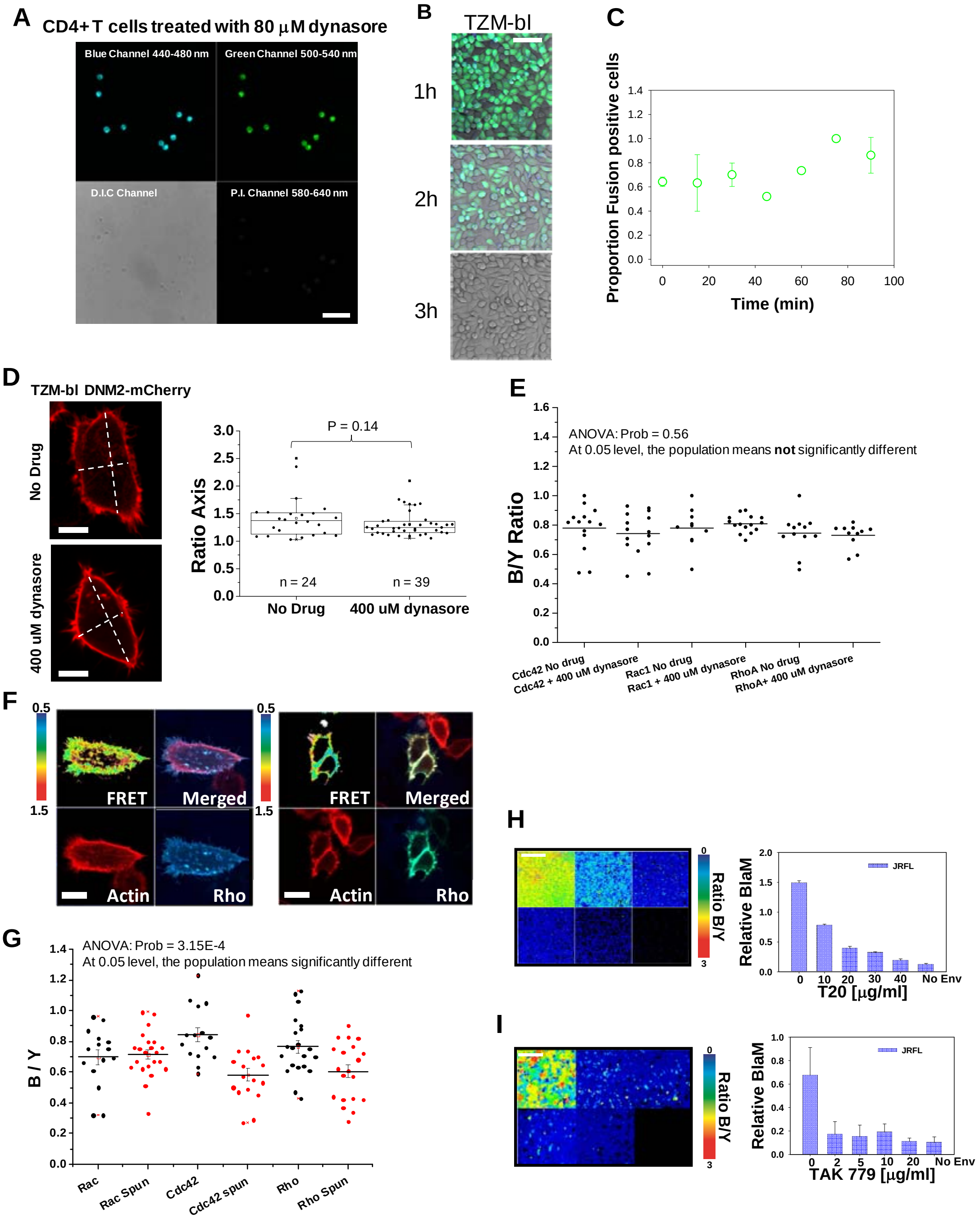


**Figure S1. Controls for Dynasore Off-side effects and cellular toxicity and spinoculation. Related to Figure 1.**

(A) CD4 T cells in the presence of 80 mM dynasore were loaded with CCF2 (blue and green channels shown) and treated with propidium iodide (PI) as described in material and methods. Scale Bar: 20 µm.
(B) CCF2 leakage in TZM-bl cells was imaged at 37 ºC and these cells turned out to leak out the sensor.Scale bar: 40 uM.
(C) Time-of-addition BlaM kinetics without spinoculation protocols on $HIV_{JRFL}$ virions using $NH_4Cl$ (open green dots).
(D) Quantitative analysis of TZM-bl cells expressing Lifeact-mCherry with and without 400 uM dynasore. The image region of interest (ROI) was set to be an ellipsoid and the area and axis of the ellipsoid recovered. The ratio of the minor and major axis was plotted as a measure of morphological changes upon addition of dynasore.
(E) TZM-bl cells expressing Cdc42, Rac1 and RhoA FRET Raichu constructs able to measure GTPase activity, were imaged utilizing FRET-based sensitized emission approach (see material and methods).
(F) TZM-bl cells co-expressing Cdc42, Rac1 and RhoA FRET Raichu constructs and Lifeact-mCherry (F) were imaged before and after applying spinoculation (i.e. spinning the cells at 4 ºC at 2100G for 30 min).
(G). The ANOVA test indicates that overall the population means were significantly different ($P = 3.15x10^4$) showing that spinoculation might induce actin re-organization and might in turn affect virus entry.
(H) Different concentrations of T20 (H) and TAK 779 were titrated using $HIV_{JRFL}$ at MOI = 1 in TZM-bl reporter cells withan end-point BlaM assay Bald particles were used as a negative control to show no-fusion conditions (last point). Fully inhibitory concentrations were found for T20 (40 ug/ml) and TAK 779 (20 ug/ml)) (I) that were utilized in the kinetic experiments.

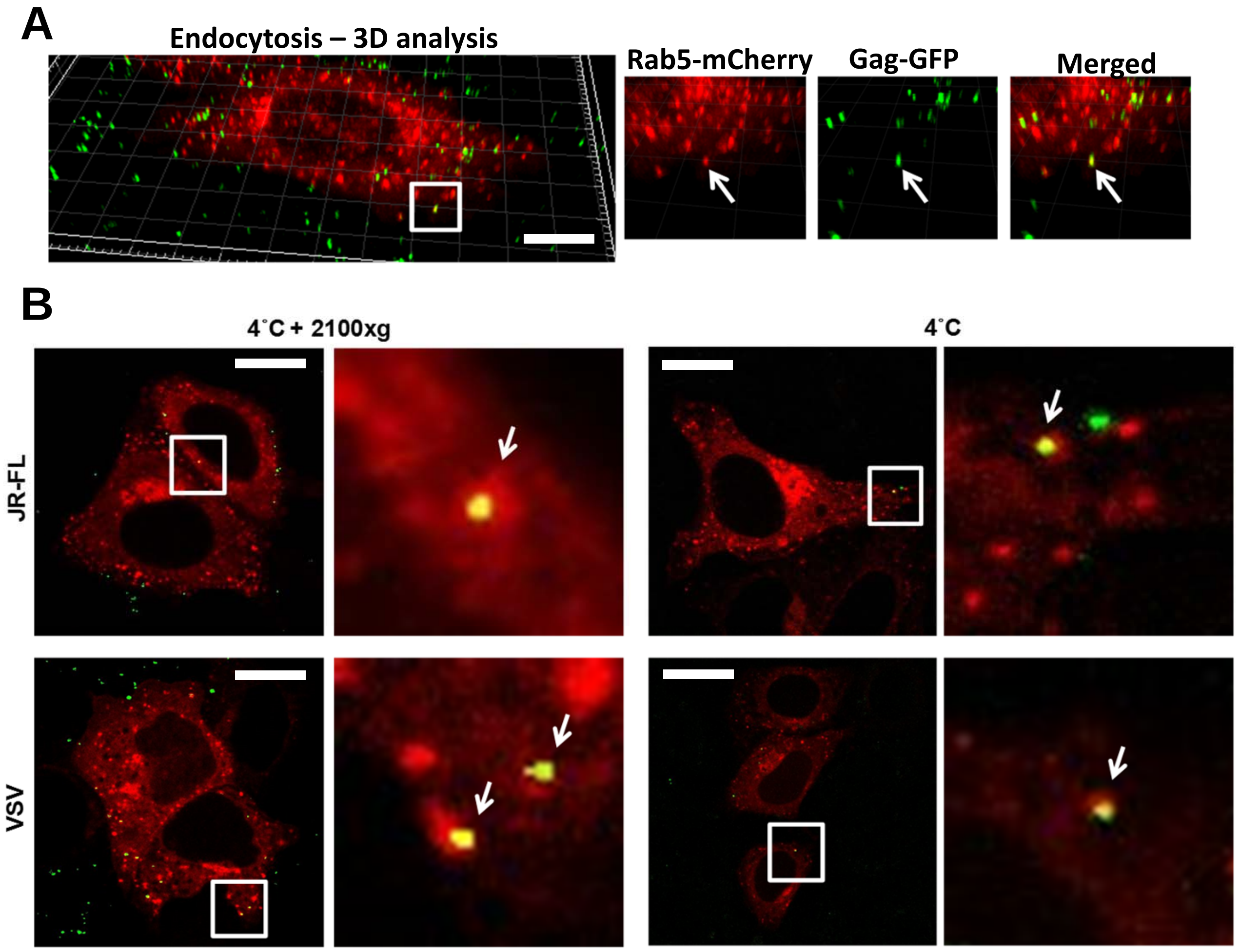


**Figure S2. The distribution of Rab5 + endosomes does not change upon addition of $HIV_{JRFL}$ or $HIV_{VSVG}$ virions. Related to Figure 2.**
(A) 3D images of TZM-bl cells expressing Rab5-mCherry exposed to high MOI (10) of HIV virions decorated with JRFL or VSVG packaging Gag-GFP were acquired with a confocal microscope (as explained in material and methods). Qualitatively the endosomes do not present different patterns. Scale bar: 10 µm. (B) HIV Gag-GFP particles colocalized with and without spinoculation protocols indicating that both virions are able to get internalized through endocytosis. Scale bars: 10 µm.

FIG. S2

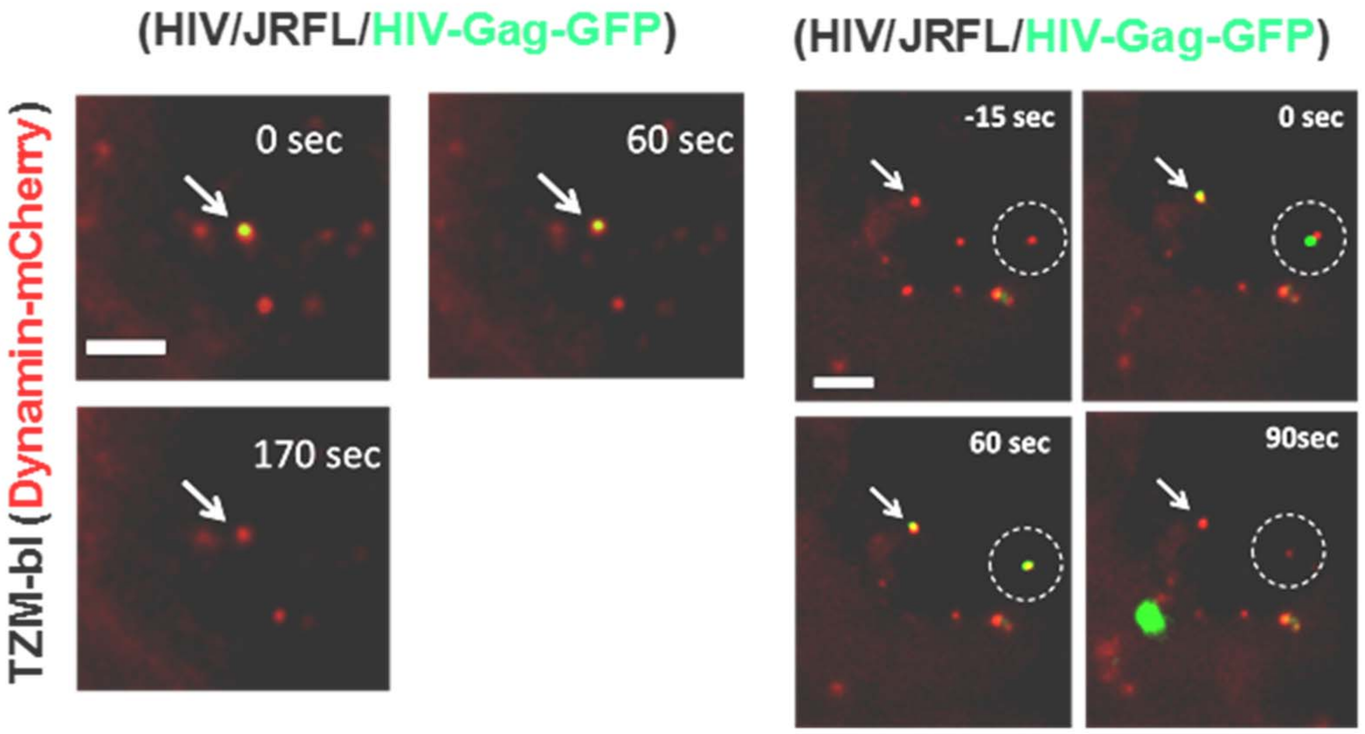


**Figure S3. Single virus tracking using TIRF shows virions able to get underneath the cell and co-localize with DNM2. Related to Figure 3.**
Labeled virions ($HIV_{JRFL}$ / Gag-GFP) were added on TZM-bl cells transfected with DNM2-mCherry and imaged with TIRF microscopy. The micrographs show virions able to get underneath the cell and co-localize with DHN2-Cherry dots (yellow dots, white arrows on the top left panel). At t = 170 s the $HIV_{JRFL}$ Gag-GFP + particles internalize or fuses whilst the DNM2-mCherry remains in the observation field. In the right panel $HIV_{JRFL}$ Gag-GFP + particles were detected getting underneath and colocalizing with DNM2-mCherry dots (white arrows and dotted circle). Scale bar: 2 µm.

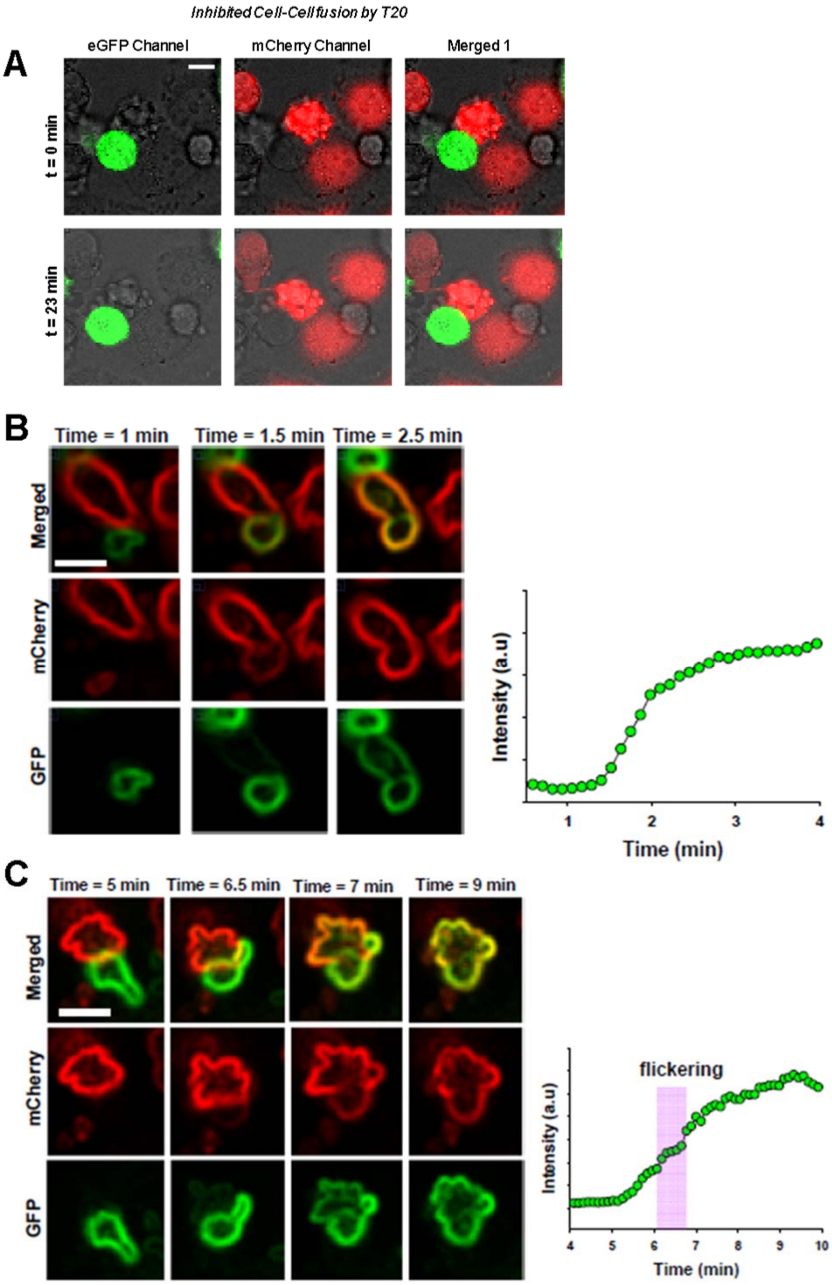


**Figure S4. Cell-cell fusion experiments. Related to Figure 4.**

(A) Composite representative micrographs depicting target and effector cells arrested by using 40 ug/uL of T20. Scale bar: 20 µm.

(B) Composite micrographs treated with a filter (as described in supplementary material and methods) depicting cell-cell fusion showing cell-cell fusion at three different time lags: 1 minutes (no fusion) and 1.5 minutes (cell-cell fusion omega formation) and full fusion (2.5 minutes). Scale bar: 15 µm.

(C) Images showing cell-cell fusion treated with 400 uM dynasore. Scale bar: 15 µm. Right panel showing the fluorescence profile recovered from the target cell in which the pink zone denotes flickering.

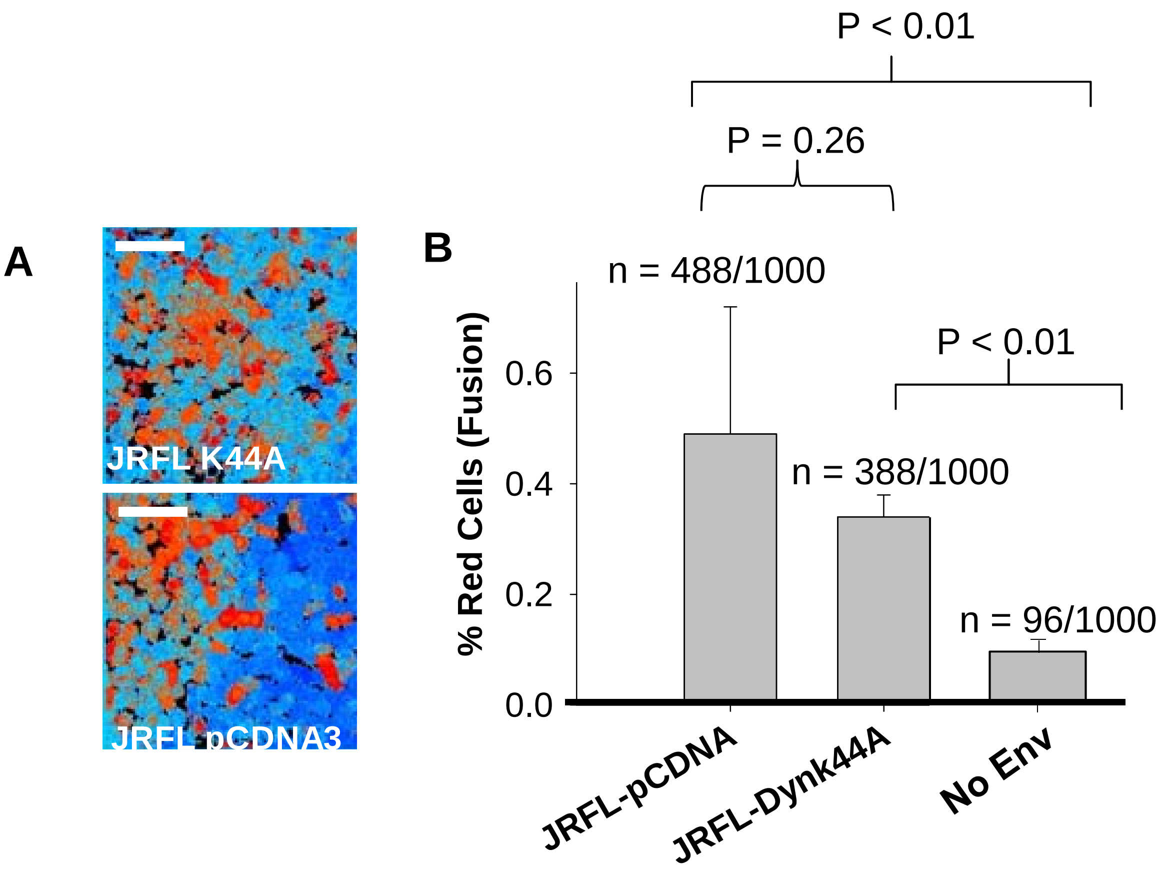


**Figure S5. HIV-1 fusion is not fully arrested in TZM-bl cells expressing dominant negative dynamin K44A. Related to Figure 5. (A)** TZM-bl cells were transfected with dynamin (K44A) and end point BlaM assays performed. Pseudocolor end-point BlaM images are presented (red cells represent $HIV_{JRFL}$ fusion positive cells and blue cells non-fusogenic cells). Scale Bar: 100 µm. (B) The proportion of fusion positive cells (% of red cells (Fusion)) is presented for each condition showing that cells transfected with an empty vector (JRFL-pCDNA) turned out to be more fusogenic for $HIV_{JRFL}$ as compared to cells transfected with dynamin (K44A), although full fusion inhibition was not attained when overexpressing dynamin (K44A).

## EXPERIMENTAL PROCEDURES

### Propidium Iodide Imaging of T cells

Propidium Iodide (PI, Thermo Fischer) is known to stain chromosomes. As it is not permeant to cells it is commonly employed to detect dead cells in a given population. We treated the CD4 T cells with 500 nM solution of PI for 30 minutes and rinsed the sample 2 times before observation under the SP8XSMD Leica confocal microscope. Of note, PI presents spectral properties that are compatible with CCF2 BlaM substrate as it is excited at 561 and its emission goes from 580 till 700 nm. We used then a WLL tuned at 561 and an hybrid detector set at 580 – 650 to collect red photons coming from dead cells. The few cells positive for PI were not considered in our Blam analysis.

### FRET- sensitized emission of Raichu constructs to measure small GTPases

The FRET Raichu constructs for Cdc42, Rac1 and RhoA were kindling provided by Matsuda lab. Briefly the GTP / GDP exchange induce a conformational change in the construct that brings together the donor (CFP) and the acceptor (YFP) fluorescent proteins. We acquired the images with a Leica SP8 utilizing a FRET-Intensity sensitized emission approach (ref). TZM-bl cells expressing each one of the Raichu biosensors were either placed directly under the microscope or underwent spinoculation protocols (spinning the cells at 2100G for 30 min). A 458 nm Argon line was used to excite the CFP and a 63X (1.3 NA) recovered the emission that was acquired using two hybrid photon counting detectors set at 460 – 500 nm (blue emission) and 520 -550 (sensitized emission + bleed through). Both channels were imaged simulateneously together with the DIC.

Pixel-by-pixel image analysis was performed by ImageJ by rationing the blue and yellow channels. When FRET occurred the yellow channel was enhanced and therefore the blue / yellow ratio was decreased (as seen in Supplementary Figure 3.

### BlaM end point analysis of cells expressing DNM2-K44A

TZM-bl cells either over-expressing DNM2 K44A mutant or not were loaded with CCF2 and excited with a 405 nm continuous laser (Leica, Manheim) and the emission spectra was set between 430-560nm and recorded pixel by pixel (512 X 512) using a Leica SP8 X-SMD microscope with a lambda resolution of 12 nm. The ratio of blue emission (440 - 480 nm, cleaved CCF2) to green (500 -540 nm, un-cleaved CCF2) was then calculated pixel by pixel

using ImageJ (http://imagej.nih.gov/ij/) for three different observation fields using a 63X objective and plotted. Fusion kinetics were then recovered with automated software (R) detecting blue/green ratios coming from individual cells above the threshold given by our negative control (No Env virions packaging Vpr-BlaM).

**Statistics and Image filtering**

All statistical analyses (one way ANOVA, hypothesis testing t-student) were performed with Originlab (Northhampton, USA). For Supplementary Figure 9 a spatial filter was applied in both channels (green and red) to focus on the edges using ImageJ ("Find Edges" algorithm, http://imagej.nih.gov/ij/).
